# Analysis of the clinical efficacy of visualization of percutaneous endoscopic lumbar discectomy combined with annulus fibrosus suture in lumbar disc herniation

**DOI:** 10.1007/s10143-023-02276-x

**Published:** 2024-01-19

**Authors:** Jintao Xi, Xiaozhen Wang, Xugui Li, Congjun Wu, Tonghui Zhang, Qilin Lu

**Affiliations:** Department of Orthopedics, The Affiliated Hospital of Wuhan Sports University, Wuhan, 430079 Hubei China

**Keywords:** Visualization of percutaneous lumbar endoscopic discectomy, Annulus fibrosus, Suture

## Abstract

The objective of this study is to compare the clinical effectiveness of visualization of percutaneous endoscopic lumbar discectomy (VPELD) combined with annulus fibrosus suture technique and simple percutaneous endoscopic lumbar discectomy (PELD) technique in the treatment of lumbar disc herniation. A retrospective analysis was conducted on 106 cases of lumbar disc herniation treated with foraminoscopic technique at our hospital from January 2020 to February 2022. Among them, 33 cases were treated with VPELD combined with annulus fibrosus suture in group A, and 73 cases were treated with PELD in group B. The preoperative and postoperative visual analogue scale (VAS), functional index (Oswestry Disability Index, ODI), healing of the annulus fibrosus, intervertebral space height, and postoperative recurrence were recorded and compared between the two groups. All patients underwent preoperative and postoperative MRI examinations, and the average follow-up period was 12 ± 2 months. Both groups showed significant improvements in postoperative VAS and ODI scores compared to the preoperative scores (*P* < 0.05), with no statistically significant difference between the groups during the same period (*P* > 0.05). There was no significant decrease in intervertebral space between the two groups after surgery (*P* > 0.05). Group A showed significantly lower postoperative recurrence rate and better annulus fibrosus healing compared to group B (*P* < 0.05). The VPELD combined with annulus fibrosus suture technique is a safe, feasible, and effective procedure for the treatment of lumbar disc herniation. When the indications are strictly adhered to, this technique can effectively reduce the postoperative recurrence rate and reoperation rate. It offers satisfactory clinical efficacy and can be considered as an alternative treatment option for eligible patients.

## Introduction

Approximately 70–85% of people experience low back pain at least once in their lifetime [1]. One of the leading causes of low back pain is lumbar disc herniation (LDH) [2]. Treatment options for LDH include conservative management and surgical interventions. Percutaneous endoscopic lumbar discectomy (PELD) has emerged as a rapidly evolving surgical technique for the treatment of lumbar disc herniations [3, 4]. PELD offers remarkable advantages, such as minimal bone resection, preservation of paravertebral muscles, rapid recovery, low procedure-related morbidity, cost-effectiveness, and high patient satisfaction rates [5]. However, similar to other spinal surgeries, PELD often involves multiple punctures under X-ray fluoroscopy and is associated with a relatively high recurrence rate, affecting up to 15% of patients [6]. Improving the success rate of surgery and reducing the recurrence rate have become key areas of focus in clinical research. Clinicians have identified a close relationship between the degree of annulus fibrosus defect and the recurrence of disc herniation through a series of studies [7]. Therefore, accompanying techniques that address the damaged annulus fibrosus and utilize targeted foraminoplasty under visualization are increasingly recognized for their potential to prevent re-herniation and enhance nucleus pulposus (NP) repair [8, 9].

The objective of the present study was to retrospectively compare the efficacy of visualization percutaneous endoscopic lumbar discectomy (VPELD) with annulus ring suture versus PELD discectomy in the treatment of LDH. Additionally, we aimed to evaluate a novel minimally invasive method for repairing the residual annulus fibrosus after discectomy, with the ultimate goal of reducing the risk of recurrence. By conducting this comparative analysis, we aimed to contribute to the advancement of treatment options for patients with LDH and improve surgical success rates.

## Patients and methods

This retrospective study included a total of 106 patients who underwent treatment for lumbar disc herniation at our hospital between January 2020 and February 2022. The study was conducted with permission from the institutional review board, and written consent was obtained from all patients.

All procedures were performed by the same surgical team routinely performed, ensuring consistency in surgical technique and approach. This is a retrospective, postoperative follow-up, and efficacy assessment of all patients was done by 2 surgeons. Analysis of the images was performed by a team of independent radiologists.

**Fig. 1:**
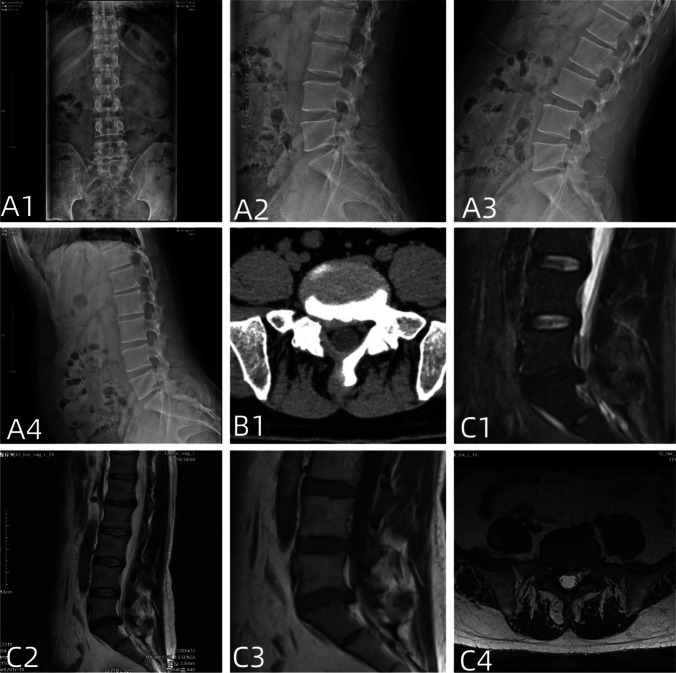
It shows the preoperative examination of a typical patient: patients **A1**–**A4** had a stable lumbar spine, **B1** had a herniated disc with no local ossification, and **C1**–**C4** had a herniated disc with an intact annulus fibrosus on magnetic resonance examination

The inclusion criteria for patient selection were as follows [10, 11]: (1) presence of definite unilateral lower limb neurogenic pain; (2) according to the MSU (Michigan State University) classification, the size-2 or size-3 lesions are selected for single-level discectomy and no significant calcification or ossification; (3) all patients were followed up for a minimum of 12 months and discharged if they remained almost asymptomatic for 6 months. If they have progressive neurological deficit and persistent back pain, were treated with surgical procedures; (4) clear identification of a single-segment responsible disc; (5) patient agreement to undergo annulus fibrosus suturing, with a relatively intact and defect-free fibrous ring after disc removal (Fig. 1).

Patients who met any of the following criteria were excluded from the study [12]: (1) presence of multi-segmental spinal stenosis, lumbar spinal slippage or instability, and spinal deformity; (2) history of previous lumbar spine surgery; (3) presence of pathological conditions such as tumor, trauma, or infection; and (4) significant calcification or ossification of the fibrous ring surrounding the ruptured disc.

All patients were informed of the advantages and disadvantages of fiber-ring suturing, and the patients chose their own surgical procedure. These criteria were applied to ensure homogeneity among the patient population and to focus on the specific parameters being evaluated in this study.

## Surgical techniques

The surgical procedure was performed under local anesthesia with the patient in the prone position on a see-through operating table. C-arm fluoroscopy was used to confirm the target segment and ensure accurate placement.

For the posterior approach, a small incision of approximately 1 cm was made slightly to the right or left of the midline of the spinous process as the skin entry point. An 18-gauge puncture needle was inserted vertically towards the vertebral hiatus. Once the needle reached the target location, the endoscopic system was installed.

For the lateral approach, the spinal coordinate direction was confirmed along the planned pathway, typically located 8–14 cm away from the midline in the posterior-lateral position. The endoscopic system was then installed.

**Fig. 2:**
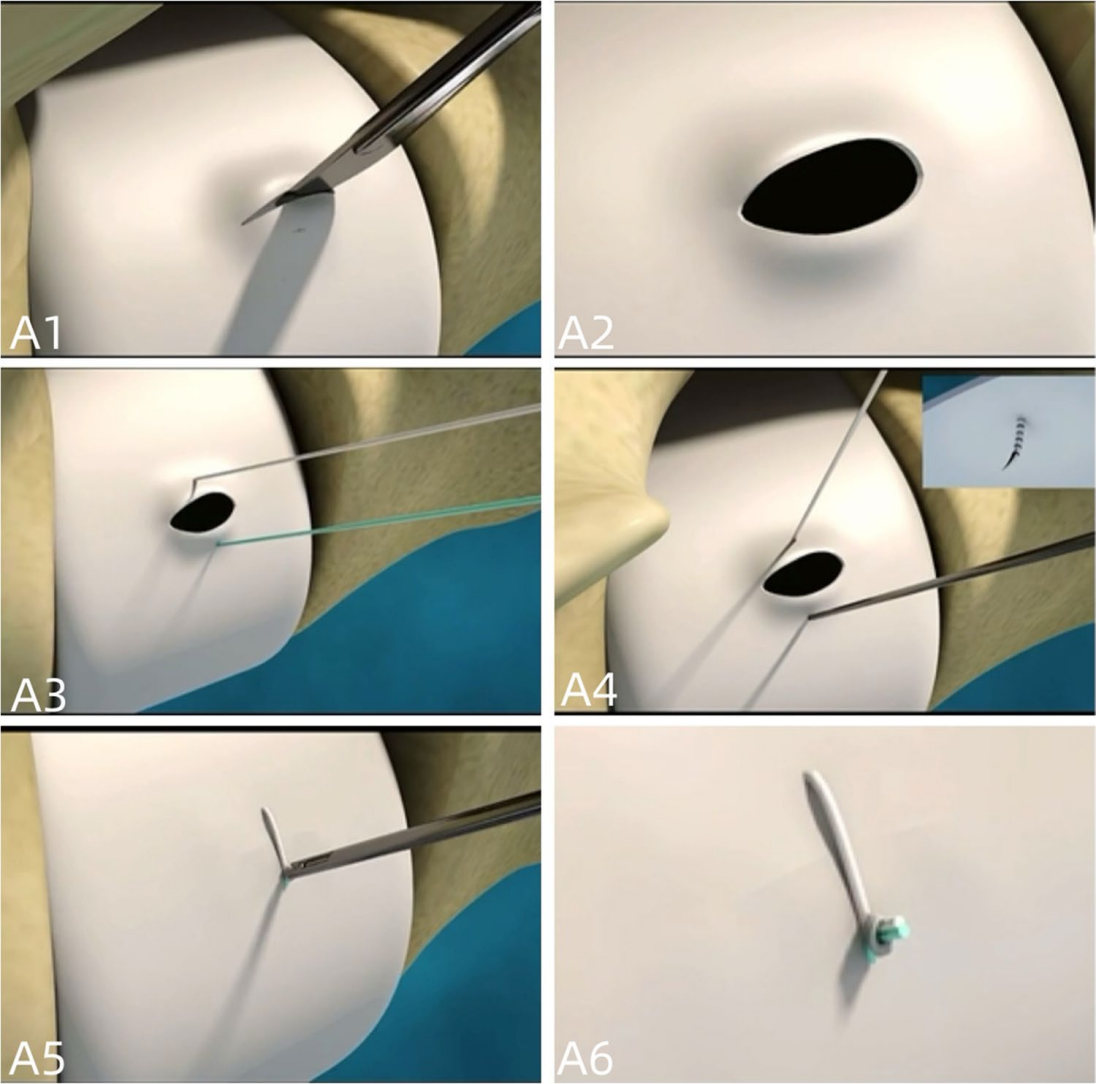
It shows the workflow of the fibrous ring suture device: **A1** incision of the fibrous ring, **A2** removal of the nucleus pulposus to completely expose the breach, **A3** piercing of the fibrous ring with a needle with thread into the breach, **A4** tensioning of the suture, **A5** tensioning of the suture for knotting, and **A6** cutting of the thread

**Fig. 3:**
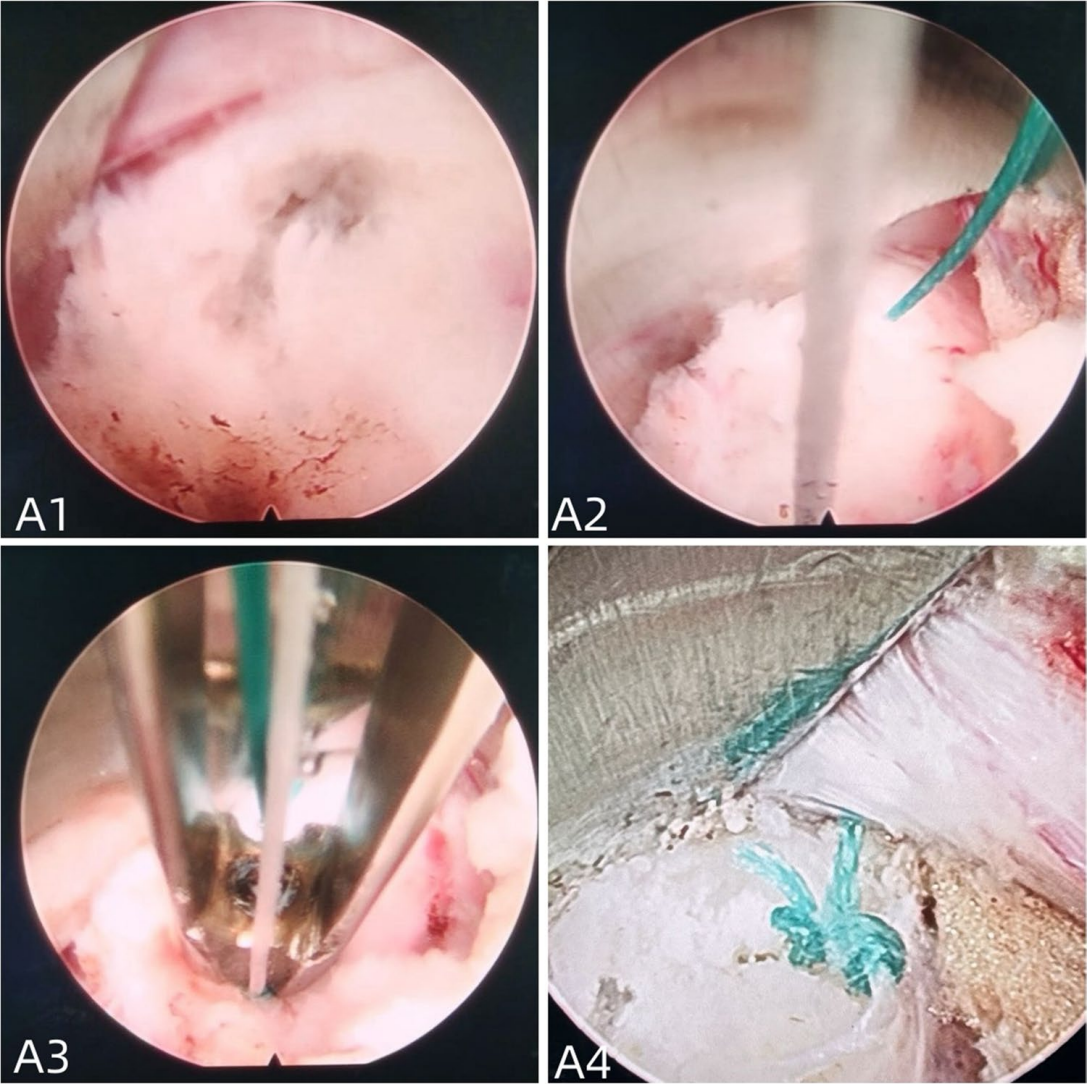
It shows the patient intraoperatively: **A1** patient with complete exposure of the fibrous ring breach, **A2** sutures placed around the breach, **A3** knotter compressing the thread, and **A4** complete suturing of the thread

During the procedure, great care was taken to separate the nerve roots from any adhesions to the disc. The herniated nucleus pulposus was carefully removed using endoscopic forceps. If there was no rupture, a longitudinal incision of approximately 5 mm was made in the middle part of the annulus fibrosus, and the protruding loose nucleus pulposus was extracted using small endoscopic forceps. The area was thoroughly checked to ensure complete removal of any residual material. Radiofrequency plasma electrode was used to shape the nucleus pulposus and annulus fibrosus, while ensuring that the posterior margin of the annulus fibrosus was smooth and the nerve root was relaxed. In cases of incision or rupture of the annulus, the annulus fibrosus was sutured using an annular suture device (Figs. 2 and 3).

These surgical techniques were employed to effectively remove the herniated disc material and repair any defects in the annulus fibrosus, thereby addressing the underlying pathology of lumbar disc herniation.

## Outcome assessment

**Fig. 4:**
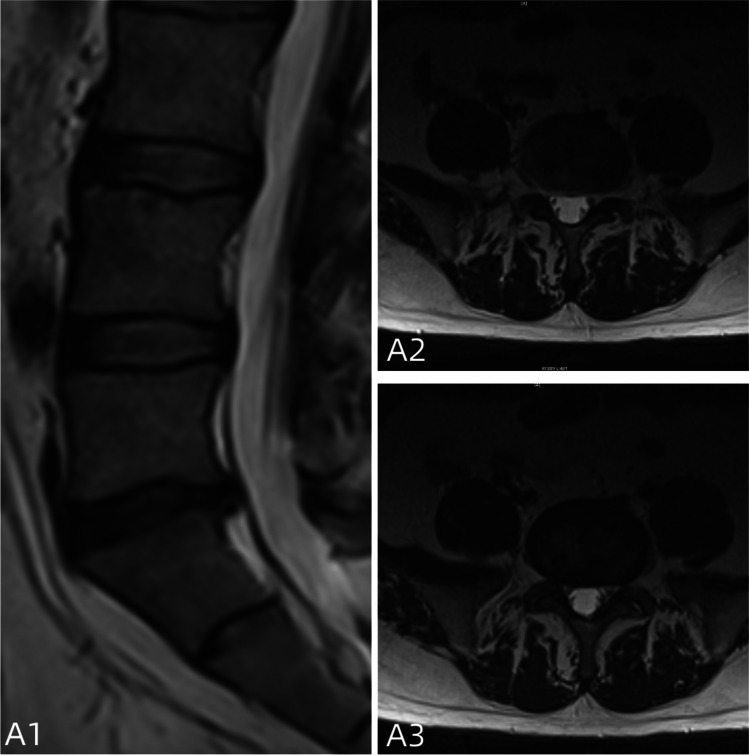
**A1**–**A3** The review 1 year after surgery; there was no significant nerve compression. Fiber ring breach is completely closed with no high signal shadow and normal intervertebral space height

**Fig. 5:**
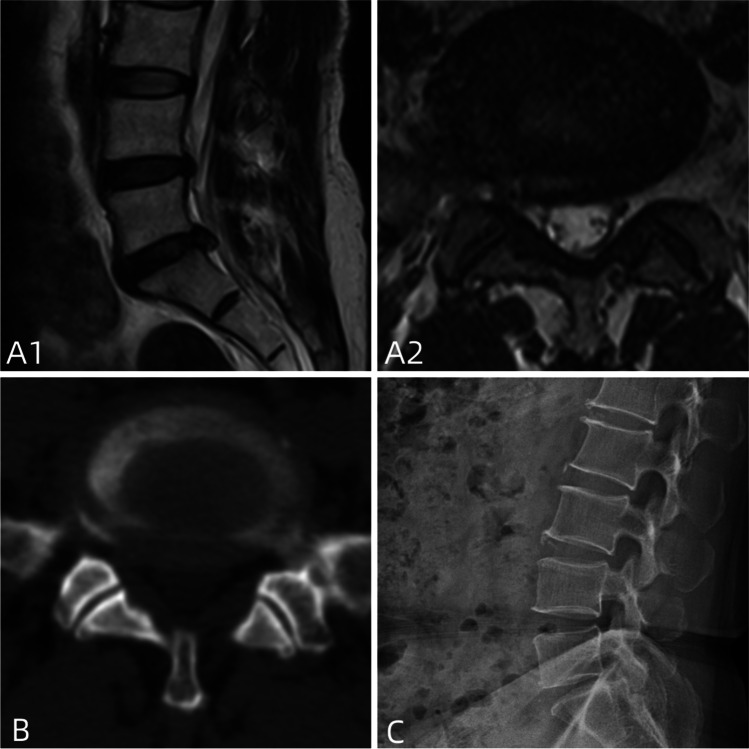
**A1**, **A2**, **B**, and **C** The preoperative examination of a typical patient (PELD without annulus fibrosus suture): patients had a stable lumbar spine and had a herniated disc with no local ossification. A herniated disc on magnetic resonance is examinated

**Fig. 6:**
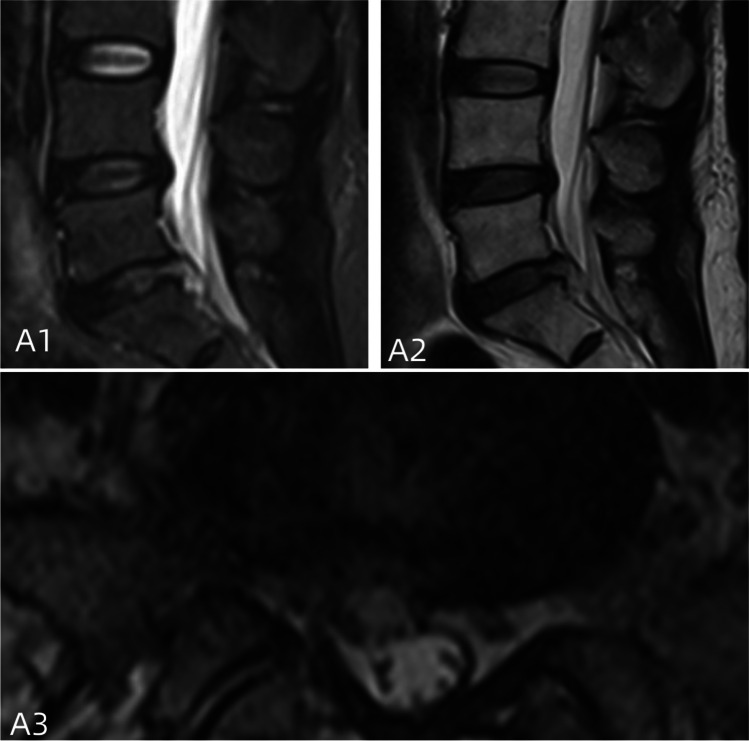
**A1**–**A3** The review 3 months after surgery; there was no significant nerve compression, but localized high signal shadow of the intervertebral disc with non-closure fibrous annulus

Postoperative assessments were conducted at 1 week, 1 month, 3 months, and 12 months. Within 1 week after surgery, MRI scans of the lumbar spine were reviewed to evaluate the completeness of herniated disc tissue removal and the adequacy of nerve decompression (Figs. 4, 5 and 6).

The following outcome measures were used: the Visual Analog Scale for back pain (VAS-back) score: a numerical scale ranging from 0 to 10 was used to assess the intensity of back pain, with higher scores indicating greater pain. The Visual Analog Scale for leg pain (VAS-leg) score: similar to the VAS-back score, this scale assessed the intensity of leg pain. The Oswestry Disability Index (ODI) score: this questionnaire-based index evaluated the impact of low back pain on daily activities and functional abilities. Scores ranged from 0 to 100, with higher scores indicating greater disability. Measurement of anterior and posterior disc heights: standing lateral radiographs were used to measure the anterior and posterior disc heights, and the average of these measurements was calculated.

The closure of the fibrous ring: MRI images from the HIS system were examined to assess the closure of the fibrous ring. Closuring criteria were classified as good (no defective high signal on continuous sagittal images), general (high signal but discontinuous), or poor (a large amount of high signal at the rupture).

Patients who met any of the following criteria were considered Lumbar disc recurrence: compared with the first review after surgery, there was obvious protrusion of the nucleus pulposus in the spinal canal and the symptoms of lower limbs were significantly worse.

## Statistical analysis

Quantitative data were presented as mean ± standard deviation. Independent 2-sample *t*-tests were used to compare clinical and radiographic records between groups. Analysis of variance (ANOVA) was employed for stratified analysis, and the chi-square test was used for comparison of categorical data. A significance level of *P* < 0.05 was considered statistically significant. Statistical analysis was conducted using SPSS software, version 17.0.

## Results

Demographic data: A total of 106 patients were included in the study. Group A consisted of 33 cases of annulus fibrosus ring sutures, with an average age of 47.81 years. Group B consisted of 73 cases of annulus fibrosus sutures, with an average age of 52.31 years. There were no significant differences in demographic data between the two groups. The demographic data is presented in Table 1.

**Table 1:** General clinical data of all patients

Variables	VPELD + annular suture group (*N* = 33)	PELD group (*N* = 73)	*P* value
Age (years)	47.81 ± 11.61	52.81 ± 9.45	*P* = 0.648 *χ*^2^ = 1.056
Gender			
Female	18	42	*P = 3.628*
Male	15	31	*t = 1.335*
Operation level			
L3/4:	10	17	*P* = *0.428* *χ*^*2*^ = *5.784*
L4/5:	13	35	
L5/S1:	10	21	
HD type			
Protrusion	5	13	*P* = 0.128 *χ*^2^ = 3.784
Herniated	25	55	
Bulge	3	5	
Operation time	52.81 ± 9.45	68.55 ± 19.37	*P* = 0.687 *t* = 1.376
The number of punctures	1.52 ± 1.08	4.5 ± 2.87	*P* = 0.001 *t* = 0.258
BMI (kg/m^2^, *x̅* ± *s*)	22.91 ± 3.61	23.66 ± 2.79	*P* = 0.071 *t* = 2.126
Surgical approach			
Lateral	24	55	*P* = 0.088 *χ*^2^ = 2.284
Posterior	9	18	

The Clinical Data Postoperative Visual Analog Scale (VAS) scores for low back and leg pain were significantly reduced in both groups compared to the preoperative scores (*P* < 0.05) (Tables 2 and 3). The Oswestry Disability Index (ODI) scores also showed improvement compared to the preoperative scores in both groups (*P* < 0.05) (Table 4). There were no significant differences in VAS and ODI scores between the two groups (*P* > 0.05). The intervertebral space height did not show significant loss in group A patients during all follow-up periods (*P* > 0.05) (Table 5). However, patients in group B had a statistically significant decrease in intervertebral space height at 3 months and the last follow-up compared to the preoperative period (Table 5). The number of punctures was significantly different between the two groups (Table 1).

**Table 2:** Comparison of VAS back pain

VAS back pain	Preop	Postop 1 week	Postop 1 month	Postop 3 months	Postop 12 months	*F*	*P*
Group A	6.07 ± 1.00	2.40 ± 0.95	1.40 ± 1.02	1.35 ± 1.07	1.00 ± 0.97	8.893	0.027^①^/0.072^②^
Group B	6.97 ± 1.30	2.56 ± 1.05	1.33 ± 1.22	1.38 ± 1.27	1.10 ± 1.07	9.965	0.048^①^/0.062^②^
*P*	0.473	0.078	0.350	0.234	0.576		
*t*	0.636	1.416	1.322	0.854	1.036

**Table 3:** Comparison of lower limb VAS scores

Lower limb VAS	Preop	Postop 1 week	Postop 1 month	Postop 3 months	Postop 12 months	*F*	*P*
Group A	6.07 ± 1.00	2.40 ± 0.95	1.40 ± 1.02	1.35 ± 1.07	1.00 ± 0.97	5.762	0.017^①^/0.062^②^
Group B	6.47 ± 1.09	2.10 ± 0.75	1.66 ± 1.32	1.15 ± 1.57	1.00 ± 1.17	6.486	0.012^①^/0.107^②^
*P*	0.084	0.119	0.532	0.641	0.738		
*t*	1.282	0.886	0.367	0.654	0.897

**Table 4:** Comparison of ODI scores among patients

ODI VAS	Preop	Postop 1 week	Postop 1 month	Postop 3 months	Postop 12 months	*F*	*P*
Group A	82.20 ± 5.06	21.00 ± 6.95	17.71 ± 2.60	14.76 ± 3.60	11.27 ± 3.36	3.880	0.001^①^/0.051^②^
Group B	78.47 ± 7.09	25.10 ± 9.75	19.15 ± 4.57	13.15 ± 6.57	11.70 ± 4.17	6.742	0.013^①^/0.077^②^
*P*	0.053	0.067	0.168	0.148	0.088		
*t*	1.636	0.486	0.252	0.322	0.354

**Table 5:** Intervertebral space height was compared between patients

Disc height	Preop	Postop 1 week	Postop 1 month	Postop 3 months	Postop 12 months	*F*	*P*
Group A	0.86 ± 0.17	0.88 ± 0.15	0.87 ± 0.07	0.81 ± 0.06	0.87 ± 0.08	0.525	0.107^①^/0.718^②^
Group B	0.76 ± 0.19	0.78 ± 0.17	0.67 ± 0.16	0.69 ± 0.06	0.67 ± 0.18	4.970	0.066^①^/0.001^②^
*P*	0.473	0.913	0.460	2.636	2.789		
*t*	0.725	− 0.109	0.747	0.013	0.010		

The radiographic data postoperative MRI scans revealed successful discectomy in all patients. The recurrence rate and reoperation rate were compared between the two groups. In group A, 1 patient experienced recurrence, resulting in a recurrence rate of 3.03%. In group B, 7 patients experienced recurrence, resulting in a recurrence rate of 9.59%. The comparison showed a significantly lower recurrence rate in group A compared to group B. Fibrous ring healing was also significantly weaker in group B compared to group A. The clinical and radiographic data are summarized in Table 6. An illustrative case is presented in Fig. 3.

**Table 6:** Comparison of postoperative recurrence between the two groups

	Recurrence probability	The repairing of annulus fibrosus
Group A	3.03%	Good: 21 case; general: 10 case; worse: 2 case
Group B	9.59%	Good: 20 case; general: 43 case; worse: 10 case
*χ*^2^	7.374	6.368
*P*	0.027	0.013

## Discussion

Lumbar disc herniation is one of the most common spinal diseases in clinical practice [13, 14]. It can clinically manifest as sciatica or radicular pain, with the most common age group affected being between 30 and 50 years [8]. The L4/5 and L5/S1 levels are the most commonly affected, although higher levels can be observed in older populations [15].

While many patients recover without surgery, some require surgical treatment for sciatica [16]. There are several treatment options available for herniation, with segmental fusion being an established method that provides satisfactory short-term results. However, these techniques are invasive, costly, and can potentially create new problems by altering the biomechanical properties of the spine, leading to adverse effects on adjacent levels [3, 17, 18]. As a result, minimally invasive techniques are becoming increasingly popular, with percutaneous endoscopic lumbar discectomy (PELD) being a representative approach. PELD is a safe procedure for soft disc herniation and causes minimal damage to the muscles and ligaments [19, 20].

**Fig. 7:**
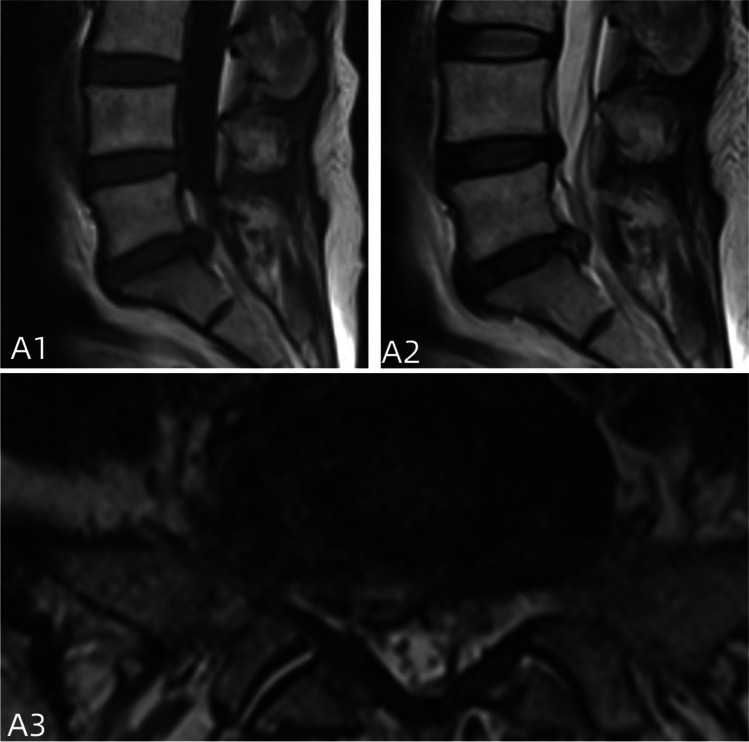
**A1**–**A3** The review 12 months after surgery; the intervertebral disc with non-closure fibrous annulus and fiber ring shows signs of recurrence

PELD involves a small incision of usually around 1.0 cm, which minimizes damage to the posterior structures and reduces interference with the nerves [19, 21]. Patients who undergo minimally invasive surgery generally experience better clinical outcomes and higher overall satisfaction [22]. However, PELD does not address the tear or fissure in the annulus fibrosus (AF) and may even exacerbate existing damage (Fig. 7). The breach in the annulus removes the protective constraints that encase and maintain pressure in the gel-like nucleus pulposus, potentially leading to reherniation. Consequently, patients who undergo minimally invasive surgery benefit from decreased pain and faster recovery but at the expense of reherniation.

The intervertebral disc is primarily composed of collagen and an inner layer rich in proteoglycans, which play crucial roles in maintaining disc viability and spinal stability [7, 23]. The AF provides tensile strength and is essential for the function of the lumbar spine [24]. However, the AF has a limited healing capacity due to its lack of significant blood supply. A defect within the AF has been shown to cause immediate and long-lasting decreases in stiffness, particularly against torsional loads [25]. If this annular defect fails to closure, it may contribute to reherniation. Successful repair of an AF defect allows for the restoration of physiological high intradiscal pressures [26]. As a result, annular repair technologies have been developed to address the inability to halt disc degeneration and decrease the risk of reherniation associated with existing techniques.

Although the fiber ring breach closure is closely related to disc recurrence after lumbar discectomy, it is important to discuss the improvement of surgical techniques for the closure or closuring of annular defects [4]. PELD involves multiple punctures under X-ray fluoroscopy, and intervertebral molding is typically performed under limited visibility. Inaccurate localization may result in repeated punctures, particularly for inexperienced surgeons. Moreover, the working channel often lacks clear markings on the body surface, which can hinder the removal of the nucleus pulposus (NP) and suturing of the fibrous ring. Previous studies have shown that even small needle punctures causing injury to the AF can accelerate disc degeneration, as evidenced by a 10-year follow-up of patients undergoing discography [27].

To improve the accuracy of location and reduce potential radiation exposure, VPELD is gradually being applied in clinical practice. One advantage of VPELD is its ability to enable targeted molding of the intervertebral foramen or lamina, thereby reducing the number of punctures and providing better visualization of annular defect morphology. In our study, we found that both PELD and VPELD + annular suture techniques achieved satisfactory postoperative pain relief and functional improvement. No significant differences were observed in postoperative outcomes between the two groups. Although the operation time and length of hospital stay were longer in the VPELD + annular suture group compared to the PELD group, these differences were not statistically significant. These findings suggest that patients may experience similar therapeutic benefits after undergoing PELD or VPELD + annular suture treatment, indicating that fiber ring sutures do not produce additional nerve stimulation.

The probability of AF closuring and recurrence was significantly lower in the VPELD + annular suture group than in the PELD group. This indicates that the fiber ring suture technique is a simple, direct, feasible, safe, and reliable surgical procedure.

The reasons for this outcome are as follows: Through annular suturing, immediate closure of the annulus fibrosus was achieved, resulting in less removal of the nucleus pulposus, which was beneficial for maintaining intervertebral space height. After 3 months, the VPELD + annular suture group showed a significantly greater disc height compared to the PELD group in our study. Maintaining disc height is important for maintaining lumbar spine stability and reducing degeneration of adjacent segments, suggesting that the annular suture technique may have better long-term effects. Yang et al. [27] demonstrated that suturing the fibers with a modified purse-string suture restored the mechanical integrity of the AF after needle puncture.

The VPELD + annular suture enables endoscopic observation of the nerve, disc, posterior longitudinal ligament, ligamentum flavum, articular eminence, and the location of the fibrous annulus. This provides a good operating space for disc removal and subsequent suturing by accurately determining the required shape, size, and position. So, AF breaks can be closed more accurately and efficiently. VPELD eliminates the need for target punctures, reducing the number of punctures and avoiding the risk of rough suture edges and decreased suture strength due to puncturing, which could affect surgical outcomes. Early fiber ring suture closure promotes immediate stability, reduces the release of inflammatory factors, and effectively alleviates back pain. Additionally, the fiber ring suture seals the rupture and prevents nerve growth, providing pain relief. Conventional surgeons often remove as much nucleus pulposus tissue as possible during surgery to minimize recurrence rates. However, this can lead to a loss of intervertebral space height and segmental instability. The fibrous ring suture technique allows for early closure of the rupture and ensures the integrity of the fibrous ring, allowing surgeons to remove as little nucleus pulposus as possible during surgery.

Although the fibrous ring is sutured, there is still a certain recurrence rate. This is mainly due to the fact that the fibrous ring has no blood supply and cannot be biologically repaired, and local scar healing can only be achieved by closing the gap with sutures. Therefore, the function is poorer than that of a normal fibrous ring, and there is still a possibility of rupture at a later stage. Large fibrous ring defects (over 6 mm), obesity, and a serious disc degeneration are also a high risk factor for postoperative recurrence [28]. These factors will result in increased stress at the site of the disc defect, which may rupture again even after suturing. It is also important for us to have a good understanding of the indications.

## Limitations

This study has several limitations. First, the sample size is small. Second, the time of follow-up for each clinical outcome was not long, which could have biased the results. Third, detailed surgical variables (e.g., the segment of operated disc levels, the type of implant used, or years of surgeon experience) were not adjusted. These variables may differ between groups and could have unknowingly impacted the results of the current study. Fourth, knowing why a given patient has a LDH is difficult. The result can be affected by genetics, lifestyle, BMI, exercise habits, or various diseases (diabetes, heart failure, chronic kidney disease, and so on). Fifth, some selection bias may exist regarding which patients undergo surgery. Unfortunately, this potential selection bias was not tracked or accounted for before the decision to undergo surgery.

## Conclusions

Our study shows promise in the use of a VPEL + annular suture for AF repair following surgical discectomy. Of course, this study is a small sample and a prospective observational study, but this study shows that VPEL + annular suture demonstrated several potential advantages, including more rapid recovery, and less recurrence rate of disc herniation.

## Data Availability

The datasets generated during and/or analyzed during the current study are available from the corresponding author on reasonable request.
